# Reactive Oxygen Species, Endoplasmic Reticulum Stress and Mitochondrial Dysfunction: The Link with Cardiac Arrhythmogenesis

**DOI:** 10.3389/fphys.2016.00313

**Published:** 2016-08-03

**Authors:** Gary Tse, Bryan P. Yan, Yin W. F. Chan, Xiao Yu Tian, Yu Huang

**Affiliations:** ^1^Li Ka Shing Faculty of Medicine, School of Biomedical Sciences, University of Hong KongHong Kong, China; ^2^Department of Medicine and Therapeutics, Faculty of Medicine, Chinese University of Hong KongHong Kong, China; ^3^Department of Epidemiology and Preventive Medicine, Monash UniversityMelbourne, VIC, Australia; ^4^Department of Psychology, School of Biological Sciences, University of CambridgeCambridge, UK; ^5^Faculty of Medicine, School of Biomedical Sciences, Chinese University of Hong KongHong Kong, China

**Keywords:** oxidative stress, reactive oxygen species, endoplasmic reticulum, mitochondria, conduction, repolarization, remodeling, arrhythmia

## Abstract

**Background:** Cardiac arrhythmias represent a significant problem globally, leading to cerebrovascular accidents, myocardial infarction, and sudden cardiac death. There is increasing evidence to suggest that increased oxidative stress from reactive oxygen species (ROS), which is elevated in conditions such as diabetes and hypertension, can lead to arrhythmogenesis.

**Method:** A literature review was undertaken to screen for articles that investigated the effects of ROS on cardiac ion channel function, remodeling and arrhythmogenesis.

**Results:** Prolonged endoplasmic reticulum stress is observed in heart failure, leading to increased production of ROS. Mitochondrial ROS, which is elevated in diabetes and hypertension, can stimulate its own production in a positive feedback loop, termed ROS-induced ROS release. Together with activation of mitochondrial inner membrane anion channels, it leads to mitochondrial depolarization. Abnormal function of these organelles can then activate downstream signaling pathways, ultimately culminating in altered function or expression of cardiac ion channels responsible for generating the cardiac action potential (AP). Vascular and cardiac endothelial cells become dysfunctional, leading to altered paracrine signaling to influence the electrophysiology of adjacent cardiomyocytes. All of these changes can in turn produce abnormalities in AP repolarization or conduction, thereby increasing likelihood of triggered activity and reentry.

**Conclusion:** ROS plays a significant role in producing arrhythmic substrate. Therapeutic strategies targeting upstream events include production of a strong reducing environment or the use of pharmacological agents that target organelle-specific proteins and ion channels. These may relieve oxidative stress and in turn prevent arrhythmic complications in patients with diabetes, hypertension, and heart failure.

## Introduction

Cardio-metabolic disorders such as diabetes mellitus and hypertension place significant burdens on the healthcare system worldwide. Over the past decades, their prevalence has been steadily increasing, due to aging and a rising level of obesity in the population (Chan and Woo, 2010; Wong et al., 2011). These disorders are leading causes of mortality and morbidity, which are traditionally attributed to cardiovascular accidents, myocardial infarction, and heart failure. Cardiac arrhythmias have increasingly been recognized as a cause of death. Diabetes may affect the prognosis of heart failure subjects differently at the clinical (Sardu et al., 2014b) and epigenetic level (Sardu et al., 2016). This article explores the normal functions of the endoplasmic reticulum (ER) and mitochondria, and synthesize current experimental data to illustrate how increased oxidative and metabolic stress can lead to dysfunction of these organelles, and in turn can initiate signaling cascades that modify the ion channel function and promote electrophysiological and structural remodeling, which ultimately promotes arrhythmogenesis.

## Normal functions of the endoplasmic reticulum, sarcoplasmic reticulum, and mitochondria

The ER is the intracellular organelle responsible for protein synthesis, folding, maturation, and assembly before they are exported to the Golgi apparatus, cytosol and plasma membrane. The oxidative environment within the lumen ensures proper formation of tertiary and quaternary structures, aided by chaperones and abundance of Ca^2+^ for interactions between them. Abnormalities in these factors can lead to unfolding or misfolding of proteins, which in turn can accumulate within the ER lumen. This would produce ER stress and elicit the unfolded protein response (UPR), which serves to reduce protein synthesis, enhance protein folding ability and aid misfolded or unfolded protein to cellular degradation pathways (Tsang et al., 2010).

The sarcoplasmic reticulum (SR) of cardiomyocytes is responsible for excitation-contraction coupling and subsequent contractile activation. Thus, dihydropyridine receptors (DIHRs) are found in the transverse tubular system and have molecular configurations that are steeply voltage-dependent, enabling them to act as voltage sensors. They are allosterically coupled to RyRs, the Ca^2+^ release channels in the SR. A depolarizing wave from the plasma membrane can spread to the transverse tubules, thereby activating the DIHRs, which then allows RyRs to dissociate from DIHRs and release the luminal Ca^2+^, inducing further release from the SR by a mechanism called Ca^2+^-induced Ca^2+^-release. This intracellular Ca^2+^ is important for initiation of contractile activity, as it binds to Ca^2+^-sensitive proteins including troponin, myosin, and actin. Relaxation occurs when intracellular Ca^2+^ returns to normal levels by following mechanisms: passive efflux mediated by the electrogenic sodium-calcium exchanger (NCX) out of the cell, active transport into the SR by sarcoplasmic reticulum Ca^2+^-ATPase (SERCA) and passive diffusion into the mitochondria via the mitochondrial Ca^2+^ uniporter (Choi et al., 2014). Finally, the SR may have functions that are traditionally ascribed to the ER, such as protein synthesis and folding (Glembotski, 2012).

The mitochondria are the site of energy supply where ATP synthesis occurs by oxidative phosphorylation, with additional roles such as regulation of apoptosis, redox status and reactive oxygen species (ROS) production (Dikalov, 2011). ATP synthesis involves electron transfer through the respiratory chain from complex I through to complex IV, culminating in protons being pumped from the mitochondrial matrix into the intermembrane space (Chance and Williams, 1955). Mitochondria are the main source of ROS, which are derived mostly from complexes I and III, as well as enzymes such as the alpha-ketoglutarate dehydrogenase complex and those involved in fatty acid beta-oxidation (St-Pierre et al., 2002; Starkov et al., 2004; Tahara et al., 2009; Brand, 2010). Oxygen free radical production is much more efficient by reverse electron transfer dependent on succinate (through complex I to NAD^+^) than forward electron transfer with NADH (Panov et al., 2007). This reverse electron transfer is an important mechanism of ROS production in many pathological conditions such as hypertension (Nazarewicz et al., 2013).

## Increased oxidative stress and intracellular organelle dysfunction in cardio-metabolic disorders

**Figure 1:**
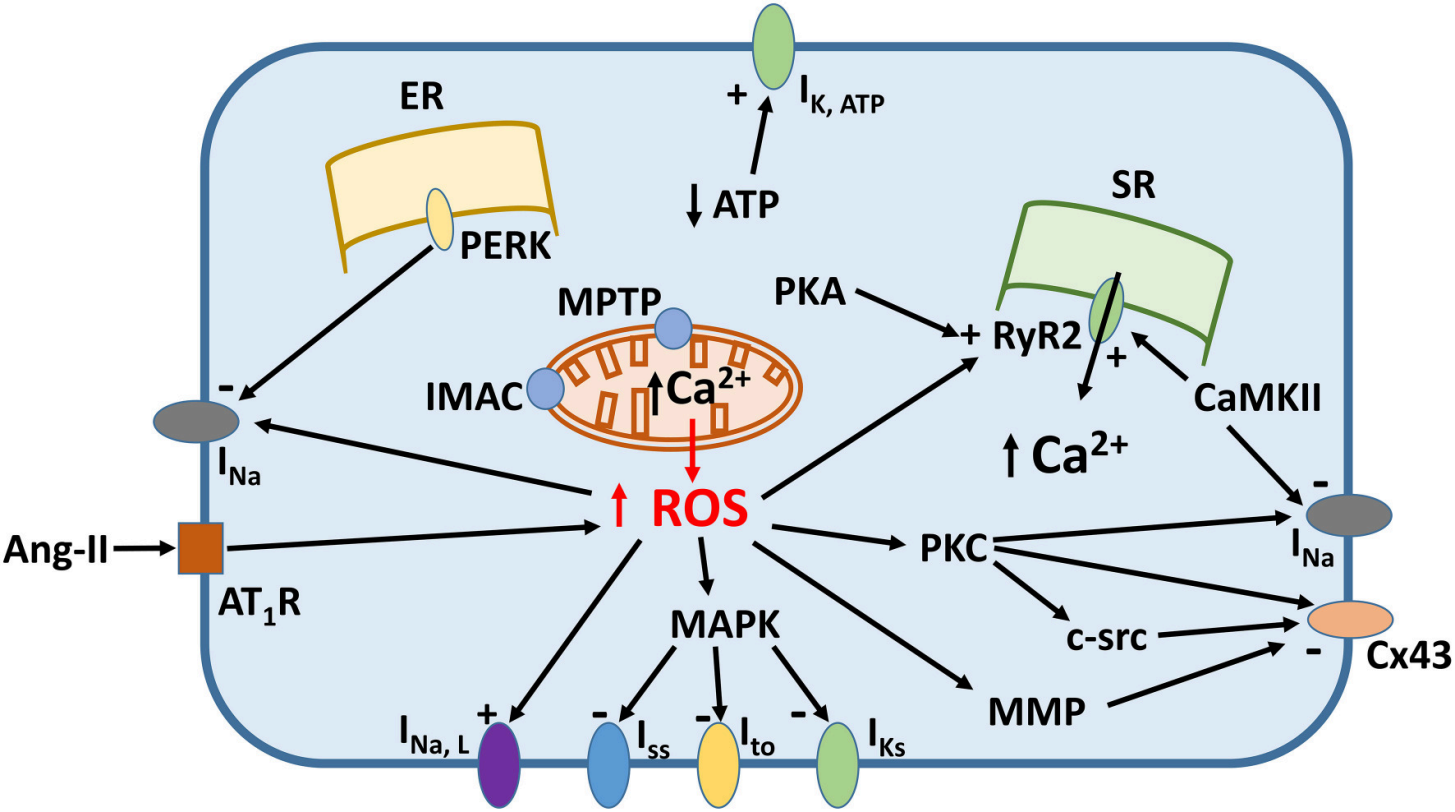
**Signaling mechanisms linking increased reactive oxygen species (ROS) production and ion channel remodeling**.

**Figure 2:**
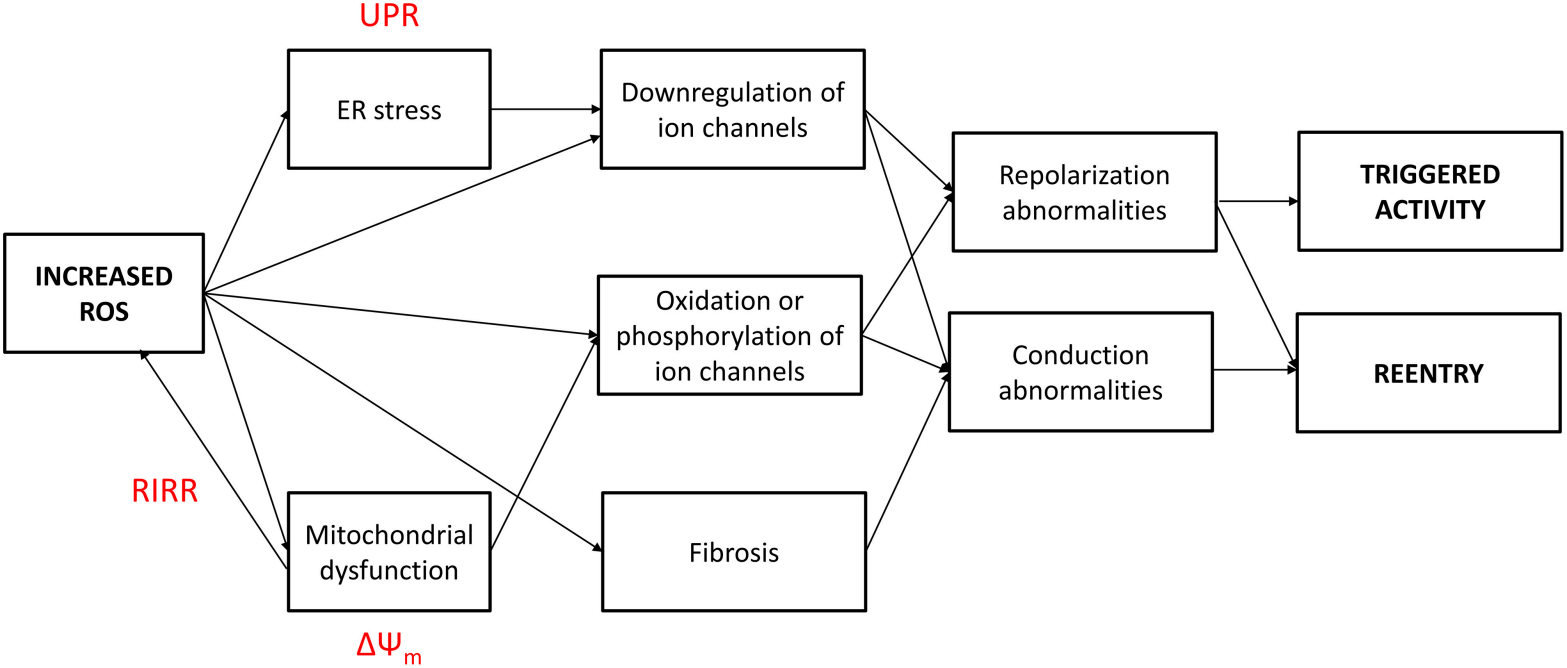
**Increased reactive oxygen species (ROS) production in cardio-metabolic disorders induce both conduction and repolarization abnormalities, thereby leading to both triggered and reentrant arrhythmogenesis**. UPR, unfolded protein response; RIRR, ROS-induced ROS release; ΔΨ_m_, mitochondrial inner membrane potential.

Increased oxidative stress from excessive ROS production appears to underlie pro-arrhythmic cardiac modeling in cardio-metabolic disorders (Figure 1). There is an increased arrhythmic burden, conditioning primary and secondary outcomes in patients with metabolic syndrome (Sardu et al., 2014a). ROS refers to superoxide, hydrogen peroxide, peroxynitrite, and hydroxyl radicals, which are unstable molecular species that can damage proteins and lipids within the cell, and activate intracellular signaling cascades. They can be generated by activation of NADPH oxidase or xanthine oxidase, uncoupling of nitric oxide synthase (NOS) or leakage of electrons from the mitochondria during oxidative phosphorylation. Normally, cardiomyocytes are protected from ROS-mediated damage by several mechanisms. Firstly, enzymes such as superoxide dismutase (SOD), catalase and glutathione peroxidase breakdown ROS into water and oxygen. Secondly, a series of redox defense systems can inactivate ROS, which are reduced glutathione (GSH), NADH, thioredoxin, and free radical scavengers such as vitamins C and E (Schafer and Buettner, 2001; Yamamoto et al., 2003; Zima et al., 2004; Gasparetto et al., 2005). Abnormal NADH accumulation, observed in diabetes, can lead to reductive stress, pseudohypoxia and subsequent, paradoxical oxidative stress; interested readers are directed to this article here (Yan, 2014). Increased oxidative stress is associated with abnormal function of intracellular organelles such as the ER and mitochondria (Wong et al., 2013; Cheang et al., 2014; Lenna et al., 2014; Murugan et al., 2015; Zhang et al., 2015). Since both the ER and mitochondria are associated with Ca^2+^ release and uptake in cardiomyocytes and abnormal Ca^2+^ handling in these cells can cause arrhythmias, it is not surprisingly that ED could be the initial trigger. ROS can also promote structural and electrophysiological remodeling, leading to abnormalities in action potential (AP) conduction or repolarization (Tse and Yeo, 2015), and in turn to triggered activity or circus-type reentry (Figure 2). These mechanisms are considered in further detail below.

## Triggered activity and reentry can result from ROS generation

### Action potential prolongation and abnormal Ca^2+^ handling can lead to triggered activity

Triggered activity results from afterdepolarizations, which are secondary depolarization events arising prematurely before generation of the next AP (Cranefield, 1977; January et al., 1991). They can occur before or after full repolarization, termed early afterdepolarizations (EADs) or delayed afterdepolarizations (DADs), respectively. EADs typically develop when action potential durations (APDs) are prolonged, due to a greater magnitude of inward depolarizing currents (*I*_Na_, *I*_Ca_, *I*_NCX_) compared to the outward repolarizing currents (*I*_Kr_, *I*_Ks_, *I*_K1_) (Tse, 2015). This would enable reactivation of the L-type calcium channel, thereby increasing *I*_Ca, L_ (January and Riddle, 1989). By contrast, DADs occur when there is intracellular Ca^2+^ overload, which induces Ca^2+^-induced Ca^2+^ release from the ER. This activates the sodium-calcium exchange current, *I*_NCX_, the nonselective cationic current, *I*_NS_ and the calcium-activated chloride current, *I*_Cl, Ca_ (Guinamard et al., 2004). The net result of EAD or DAD is membrane depolarization and initiation of another AP. If the afterdepolarizations are not sufficiently large to induce an AP, they can exacerbate the regional differences in repolarization, which may lead to alternans, unidirectional conduction block and reentry (Tse et al., in press).

Electrophysiological remodeling is observed in heart failure, with mostly downregulation of ion channels, leading to APD prolongation and/or altered calcium release, predisposing to EADs, DADs, and reentry (Marfella et al., 2013; Tse et al., 2016a). Moreover, the activities of ion channels may condition heart failure and arrhythmic state, regulating epigenetic mechanisms related to cardiac adaptive responses (Sardu et al., 2014c, 2015). The renin-angiotensin system (RAS) becomes activated in diabetes, hypertension and heart failure. This pathway is responsible for enzymatic cleavage of angiotensinogen, which is converted to angiotensin I and angiotensin II (Ang-II) by renin and angiotensin converting enzyme (ACE), respectively. Ang-II can activate the AT_1_ receptor, which increases NADPH oxidase activity to promote ROS production, resulting in downregulation of the calcium-independent transient outward K^+^ channel (*I*_to_) (Lebeche et al., 2006). ROS can increase the activity of MAPK-activated protein kinase-1 (p90^rsk^), which phosphorylates and decreases the activity of *I*_to, f_, *I*_K, slow_ and *I*_SS_ channels (Lu et al., 2008). ROS can prolongs the late sodium current, *I*_Na, L_ (Ma et al., 2005; Song Y. et al., 2006).

Moreover, prolonged activation of UPR during ER stress in heart failure leads to increased oxidative stress, downregulation of *I*_to_ and increased Ca^2+^ release from the SR. RyR2, the cardiac-specific isoform, is affected by post-translational modification from intracellular signaling cascades. Thus, they can be oxidized by ROS or reactive carbonyl species (RCS) (Eager et al., 1997; Xu et al., 1998; Bidasee et al., 2003), or phosphorylation by protein kinases such as protein kinase A or Ca^2+^/calmodulin-dependent protein kinase II (CAMKII), leading to abnormal gating (Witcher et al., 1991; Hain et al., 1995; Wehrens et al., 2004). This in turn leads to reduced amplitude but increased frequency of Ca^2+^ sparks and increased RyR2 sensitivity to Ca^2+^ activation (Shao et al., 2007, 2009). RyR2 decoupling from LTCCs can lead to desynchronized Ca^2+^ release (Song L.S. et al., 2006). ROS derived from the mitochondria could inhibit SERCA and stimulate RyR2, thereby activating NCX, Ca^2+^-sensitive cationic channels and Ca^2+^-induced Ca^2+^-release (Li et al., 2015).

Aside from regulation of energy production, mitochondria can also take up and release Ca^2+^ through several mechanisms, and may therefore serve as a buffer system. It may be that under physiological conditions, mitochondria do not have a major role in buffering Ca^2+^ (O'Rourke and Blatter, 2009; Williams et al., 2013). However, in heart failure, there is significant Ca^2+^ overload in both the cytoplasm and the mitochondria of cardiomyocytes (Santulli et al., 2015b). Experiments in a mouse model of heart failure generated by myocardial infarction, leaky RyR2 was shown to be responsible for mitochondrial Ca^2+^ overload (Santulli et al., 2015b). This would lead to mitochondrial dysfunction and increased ROS production. A recent study found that in mice with RyR2 mutations leading to diastolic Ca^2+^ leak, increased RyR2 oxidation, mitochondrial dysfunction and increased ROS production are observed, and these findings were associated with an increase in atrial fibrillation (AF) susceptibility (Xie et al., 2015a). Other studies also support the critical role of increased oxidative stress in the development and maintenance of AF. Thus, ROS from the atria correlated with the duration and substrate of AF in goats and humans (Reilly et al., 2011). Increased mitochondrial oxidative stress leading to RyR2 leak has also been observed in the ventricles of a peroxisome proliferator-activated receptor-γ (PPARg) cardiac overexpression mouse model (Joseph et al., 2016). The net electrophysiological effect was AP prolongation and development of ectopic activity.

Together, all of the above mechanisms can prolong APD and/or lead to abnormal Ca^2+^ release, inducing EADs and DADs and therefore triggered activity.

### Disruption of normal AP conduction or repolarization and abnormal Ca^2+^ handling can lead to reentry

The conduction of cardiac APs through the working myocardium has traditionally been described by the core conductor model. This is based on the cable theory, whose equations can be derived by applying Kirchoff's circuit law. Conduction velocity (CV) is determined by both passive and active properties of the cell membrane. The passive components are axial resistance of the myoplasm (r_i_), resistance of the extracellular space (r_o_), and membrane capacitance (C_m_). Diameter of the cardiomyocyte is important because it alters both r_i_ and C_m_. Active membrane properties refer to the voltage-gated conductances, mainly the sodium current (*I*_Na_) mediating the AP upstroke. This model was subsequently modified to account for the presence of gap junctions (Saffitz et al., 1994; Rohr, 2004). Each gap junction consists of two connexons, which are hexameric proteins of the connexin (Cx) subunit, mediating electrical communication by electrotonic spread. However, it forms a high resistance pathway that can decrease CV and produce discontinuous propagation (Spach et al., 1981; Spach, 2003). Its expression is not equally distributed across the myocardium, higher numbers are found at the ends of myocytes compared to the lateral aspects of the cell membrane (Kumar and Gilula, 1996). Consequently, CV is higher in the longitudinal direction than in the transverse direction, which is termed anisotropic conduction (Sano et al., 1959; Clerc, 1976). The role of ephaptic coupling has been neglected until recently, involving a direct electrical field mechanism for mediating conduction (Rhett and Gourdie, 2012; Lin and Keener, 2013; Rhett et al., 2013; Veeraraghavan et al., 2014a,b,c; George et al., 2015; Veeraraghavan et al., 2015). Finally, myocardial fibrosis cam reduce the coupling between cardiomyocytes, increasing r_i_, and enhance the coupling between fibroblasts and cardiomyocytes (Camelliti et al., 2004; Chilton et al., 2007), increasing c_m_, both of which would reduce CV and increase the dispersion of CV (Tse and Yeo, 2015).

Circus-type reentry occurs when an AP travels around an obstacle and re-excites its site of origin, and requires both conduction and repolarization abnormalities (Tse, 2015; Tse et al., 2016d,h). Firstly, conduction velocity (CV) must be sufficiently slowed to prevent the AP depolarization wavefront from colliding with its repolarization wave back, where the local myocardium is still in its effective refractory period (ERP) and cannot be excited. If this occurs, conduction will be blocked and the AP will extinguish, resulting in a break of the reentrant circuit. Secondly, unidirectional conduction block must be present. Otherwise, wavefront APs traveling in opposite directions can collide and extinguish, thereby terminating the arrhythmia. Thirdly, an obstacle can be a non-conducting or refractory region, arising from structural defects such as interstitial fibrosis, or dynamically from ectopic activity. When the excitation wavelength of the AP wave (λ, CV × ERP) is smaller than the path length, reentry can occur. The ERP usually coincides with the APD. Thus, reduction in CV, APD, or ERP can promote reentry by reducing λ, whereas increased λ reduces the likelihood of reentry (Tse et al., 2012; Choy et al., 2016; Tse, 2016b; Tse et al., 2016c,e,f,g,h,i,k,l).

All of the above factors are influenced by ROS, altering ion channel function and promoting extracellular matrix (ECM) remodeling, which together would produce conduction and repolarization abnormalities, thereby leading to reentry (Tse and Yeo, 2015). For example, the SCN5A gene encoding for the cardiac Na^+^ channels is regulated by the transcription factor NF-κB (Shang and Dudley, 2005). Elevated angiotensin II levels and the increased oxidative stress increase NF-κB binding to the SCN5A promoter region, thereby decreasing its transcriptional activity (Shang et al., 2008). Ang-II can also downregulate Cx43 and Cx40, reducing intercellular coupling (Kasi et al., 2007). During ER stress, PERK activation downregulates the Na^+^ channels, although this is a non-ion channel specific effect since *I*_to_ expression is also reduced (Gao et al., 2013). Furthermore, abnormal Ca^2+^ release can have direct and indirect effects. Ca^2+^ can bind directly to the evolutionarily conserved EF hand motif at the carboxyl end of the Na^+^ channel, thereby producing a depolarizing shift in its voltage-dependent inactivation, which prolongs the late sodium current, *I*_Na, L_ (Wingo et al., 2004; Song Y. et al., 2006). Moreover, the Na^+^ channel also possesses an IQ domain for Ca^2+^/ CaM binding and additionally several serine/threonine residues amenable to phosphorylation, for example by CaMKII (Ashpole et al., 2012), leading to channel inactivation (Tan et al., 2002).

Ca^2+^ can activate protein kinase C (PKC) (Luo and Weinstein, 1993), which can phosphorylate both Na^+^ channels (Qu et al., 1996) and Cx43 (Moreno et al., 1994; Kwak et al., 1995) to reduce *I*_Na_ and *I*_gap_, respectively. Ca^2+^ overload is also associated with dephosphorylation of gap junctions (Huang et al., 1999), leading to their uncoupling (Beardslee et al., 2000) and lateralization (Smith et al., 1991; Lampe et al., 2000). Interestingly, a recent report demonstrated that streptozotocin-induced diabetic mice have increased phosphorylation levels of Cx43 at the serine 262 residue (Palatinus and Gourdie, 2016). Previous experiments showed that Ser 262 phosphorylation of Cx43 is associated with a cardiac injury-resistant state (Srisakuldee et al., 2009). Poor myocardial healing could increase the likelihood of conduction abnormalities, which would increase arrhythmogenicity. Finally, homocysteine, which is raised in hypertension and diabetes, causes EEC dysfunction and apoptosis (Miller et al., 2000; Wei et al., 2010), suppression of superoxide dismutase and activation of matrix metalloproteinases (MMPs) in the plasma membrane, causing Cx43 disruption (Rosenberger et al., 2006). Increased Ca^2+^ leak through the RyR2 channels results in Ca^2+^ alternans, which in turn can drive APD alternans and reentry (Xie et al., 2013).

Mitochondrial ROS production is elevated in diabetes and hypertension (Kakkar et al., 1995; Kanazawa et al., 2002; Dikalov and Ungvari, 2013). Mitochondrial ROS can itself increase its own production in a positive feedback loop, termed ROS-induced ROS release (RIRR), a mechanism recently recognized as a key initiator of mitochondrial depolarization (Zorov et al., 2000). Thus, Ang-II can activate the AT_1_ receptor, which stimulates Nox2, an enzyme that catalyzes electron transfer from NADPH to oxygen, generating oxygen free radicals and hydrogen peroxide. The oxygen free radical can enter the mitochondria and activate PKCε at the mitochondrial inner membrane (Jaburek et al., 2006). The downstream target is mitochondrial K_ATP_ channels (mitoK_ATP_), resulting in reverse electron transfer from complex II to complex I, which generates more oxygen free radicals (Dikalov et al., 2014). The latter can be converted to hydrogen peroxide, activating c-src (Aikawa et al., 1997) and in turn further activating NADPH oxidases, production of free radicals and NOS uncoupling. Mitochondrial K_ATP_ channel activation in cardiomyocytes in diabetes led to impaired APD adaptation, which promoted the occurrence of VT (Xie et al., 2015b). Ang-II mediated mitochondrial ROS production can lead to c-src activation and reduced Cx43 expression (Sovari et al., 2013). Homocysteine can activate mitochondrial MMPs, which leads to opening the mitochondrial permeability transition pore (MPTP) (Moshal et al., 2008; Montaigne et al., 2013) and collapse of the mitochondrial inner membrane potential (ΔΨ_m_) (Brown et al., 2010).

ROS can also activate the mitochondrial inner membrane anion channels (IMAC) to result in mitochondrial depolarization (Yang et al., 2010). RIRR-induced regional mitochondrial depolarization results in the formation of a metabolic sink (Zhou et al., 2014). Uncoupling of oxidative phosphorylation can reverse mitochondrial ATP synthase, thereby depleting intracellular ATP. This causes sarcolemmal K_ATP_ activation, increased K^+^ efflux and APD shortening (Garlid et al., 1997; Zhou et al., 2014). Moreover, ROS can promote myocardial fibrosis via a NOX4-mediated, ROS-ERK1/2-MAP Kinase-dependent mechanism (Aragno et al., 2006; Kuroda et al., 2010).

Together, the above factors can lead to reduction in CV, together with increased repolarization gradients, can promote unidirectional conduction block and reentry (Tse et al., in press).

## Endothelial-cardiomyocyte coupling, paracrine signaling, and arrhythmogenesis

Endothelial cells form the inner lining of blood vessels, thereby controlling vascular tone by releasing vasodilators, such as nitric oxide (NO), prostacyclin and endothelial-dependent hyperpolarizing factor. In the heart, cardiac endothelial cells (CECs), including myocardial coronary endothelial cells (MCECs) lining the coronary microvasaculature and endocardial endothelial cells (EECs), outnumber cardiomyocytes (CM) with a 3:1 ratio and have roles distinct from those of the vascular endothelium, such as regulation of cardiomyocyte growth and modulation of electrical and mechanical functions (Baldwin and Artman, 1998; Brutsaert, 2003). They may do so in a paracrine manner by secreting messengers such as NO, prostaglandins, endothelin and angiotensin II. These may act on specific receptors on the plasma membrane of cardiomyocytes (e.g., Ang-II binding to AT_1_R), diffuse through the membrane of cardiomyocytes (in the case of gasotransmitters such as NO), or enter via gap junctions, to activate their downstream effector. Gap junctions are non-specific pores that also allows the spread of molecules up to 1 kDa in molecular mass (Harris, 2001; Weber et al., 2004). However, MCECs lack gap junctions and their communication with surrounding cells occur by other mechanisms.

Endothelial dysfunction is a hallmark of diabetes mellitus, hypertension and heart disease, playing a role in the initiation and progression of vascular dysfunction, eventually resulting in atherosclerosis, thrombosis and arrhythmogenesis (Wong et al., 2013). This usually refers to dysfunction of vascular endothelial cells that include the MCECs. However, EECs also become dysfunctional in diabetes and hypertension (Chu et al., 1995; Popov et al., 1996; Brutsaert, 2003). These could initiate signaling cascades that ultimately modify ion channel function and promote electrophysiological and structural remodeling in the myocardium to produce an arrhythmic substrate (Miller et al., 2002; Rosenberger et al., 2006; Moshal et al., 2008; Givvimani et al., 2011).

## Clinical relevance and future perspectives

Reduction in oxidative stress is an attractive strategy for preventing ROS-induced ion channel dysfunction, electrophysiological and structural remodeling. In keeping with this theory, exogenous application of strong reducing agents such as hydrogen sulfide was shown to reduce cardiac fibrosis. Increasing the expression of enzymes breaking down free radicals, such as SOD (Dikalova et al., 2010). Glutathione peroxidase overexpression in diabetic rat hearts prevented adverse structural remodeling by reducing fibrosis and improving diastolic dysfunction (Matsushima et al., 2006). This would be expected to reduce the degree of conduction abnormalities and thereby increase the threshold for arrhythmogenesis.

Much of the cellular oxidative stress is derived from the mitochondria, which promotes arrhythmogenesis (Xie et al., 2015a). Therefore, antioxidants specifically targeting this organelle would be a logical strategy for anti-arrhythmic therapy. Thus, experiments in genetically modified, ACE8/8 mice with RAS activation have demonstrated therapeutic effects of a mitochondrial-specific antioxidant, but not a general antioxidant, in preventing ventricular arrhythmias (Sovari et al., 2013). Altered mitochondrial bioenergetics characterized by mitochondrial depolarization is a key initiating factor of an adverse positive feedback loop of causing RIRR. Interestingly, inhibition of the MPTP by cyclosporine A did not prevent the collapse of ΔΨ_m_ (Berkich et al., 2003), whereas IMAC inhibition using the mitochondrial benzodiazepine receptor did (Akar et al., 2005). Recent work has demonstrated that patients with catecholaminergic polymorphic VT, in whom the RyR2 is mutated and leaky, insulin secretion and glucose metabolism were dysregulated (Santulli et al., 2015a). Since mitochondrial Ca^2+^ overload is due to a leaky RyR2 (Santulli et al., 2015b), ryanodine receptor stabilizers can be used to reduce this leak and reduce arrhythmic burden (Lehnart et al., 2006). ER stress is also a potential target, since a prolonged UPR can induce adverse electrophysiological remodeling. For example, overexpression of chaperone proteins Grp78 and Grp94 can increase binding to UPR sensors and abnormal proteins, in turn alleviating ER and oxidative stress as well as Ca^2+^ overload (Vitadello et al., 2003; Pan et al., 2004).

Many of the above findings have been derived from animal models, which are amenable to genetic and pharmacological manipulation (Tse et al., 2012; Chen et al., 2016; Tse et al., 2016b,c,e,f,g,k, in press). In these systems, electrophysiological consequences of ion channel mutation or dysfunction can be examined using different recording methods such as monophasic action potential or bipolar electrogram recordings, and optical mapping (Vigmond and Bardakjian, 1995; Vigmond and Leon, 1999; Vigmond, 2005; Vigmond et al., 2009; Tse et al., 2016j). Furthermore, detection of magnetic signals has been used in clinical practice: for example magnetic resonance imaging is excellent for characterization of cardiac structures in humans but are less practical for use in animal studies (Vassiliou et al., 2014; Tse et al., 2015a,b). Magnetocardiography (MCG) can be used to diagnose and predict the risk of cardiac arrhythmias in humans (Steinhoff et al., 2004; Sato et al., 2012; Kwong et al., 2013; Ito et al., 2014; Yoshida et al., 2015). A micromagnetometer array with a superconducting quantum interference device (SQUID) can be used for non-contact recording of MCGs from mice and has the potential for high-throughput use to screen for pro- or anti-arrhythmic effects of pharmacological agents (Komamura et al., 2008). Given the evidence presented here, it is clear that increased ROS can lead to intracellular organelle dysfunction and arrhythmias in cardio-metabolic disorders such as diabetes mellitus and hypertension. It is therefore prudent that the at-risk population is identified, which would enable early intervention to reduce arrhythmic mortality (Cardoso et al., 2003; Salles et al., 2005; Stettler et al., 2007; Clemente et al., 2012; Tse, 2016a,c; Tse and Yan, 2016a,b).

Nevertheless, because of the complex nature of cardiac spatiotemporal dynamics, a systems approach is needed to determine the net effect of a pharmacological agent on arrhythmogenicity. Identification of the molecular events is important for developing therapeutic strategies to reduce oxidative stress, which could slow or even reverse disease progression of cardio-metabolic disorders, which would in turn reduce the risk of cardiac arrhythmias complicating these conditions in this patient population.

## Author contributions

GT: Design of manuscript; drafted and critically revised the manuscript for important intellectual content; preparation of figures. BY: Interpreted primary research papers; critically revised the manuscript for important intellectual content. YC: Critically revised the manuscript for important intellectual content. XT and YH: Drafted and critically revised the manuscript for important intellectual content.

### Conflict of interest statement

The authors declare that the research was conducted in the absence of any commercial or financial relationships that could be construed as a potential conflict of interest. The reviewer QY and handling Editor declared their shared affiliation, and the handling Editor states that the process nevertheless met the standards of a fair and objective review.
